# Are Epithelial Ovarian Cancers of the Mesenchymal Subtype Actually Intraperitoneal Metastases to the Ovary?

**DOI:** 10.3389/fcell.2020.00647

**Published:** 2020-07-17

**Authors:** Ye Hu, Barbie Taylor-Harding, Yael Raz, Marcela Haro, Maria Sol Recouvreux, Enes Taylan, Jenny Lester, Joshua Millstein, Ann E. Walts, Beth Y. Karlan, Sandra Orsulic

**Affiliations:** ^1^Department of Obstetrics and Gynecology, David Geffen School of Medicine, University of California, Los Angeles, Los Angeles, CA, United States; ^2^Women’s Cancer Program at the Samuel Oschin Comprehensive Cancer Institute, Cedars-Sinai Medical Center, Los Angeles, CA, United States; ^3^Division of Biostatistics, Department of Preventive Medicine, Keck School of Medicine, University of Southern California, Los Angeles, CA, United States; ^4^Department of Pathology and Laboratory Medicine, Cedars-Sinai Medical Center, Los Angeles, CA, United States; ^5^Jonsson Comprehensive Cancer Center, University of California, Los Angeles, Los Angeles, CA, United States

**Keywords:** ovarian cancer, mesenchymal, molecular subtype, metastases, desmoplasia

## Abstract

Primary ovarian high-grade serous carcinoma (HGSC) has been classified into 4 molecular subtypes: Immunoreactive, Proliferative, Differentiated, and Mesenchymal (Mes), of which the Mes subtype (Mes-HGSC) is associated with the worst clinical outcomes. We propose that Mes-HGSC comprise clusters of cancer and associated stromal cells that detached from tumors in the upper abdomen/omentum and disseminated in the peritoneal cavity, including to the ovary. Using comparative analyses of multiple transcriptomic data sets, we provide the following evidence that the phenotype of Mes-HGSC matches the phenotype of tumors in the upper abdomen/omentum: (1) irrespective of the primary ovarian HGSC molecular subtype, matched upper abdominal/omental metastases were typically of the Mes subtype, (2) the Mes subtype was present at the ovarian site only in patients with concurrent upper abdominal/omental metastases and not in those with HGSC confined to the ovary, and (3) ovarian Mes-HGSC had an expression profile characteristic of stromal cells in the upper abdominal/omental metastases. We suggest that ovarian Mes-HGSC signifies advanced intraperitoneal tumor dissemination to the ovary rather than a subtype of primary ovarian HGSC. This is consistent with the presence of upper abdominal/omental disease, suboptimal debulking, and worst survival previously reported in patients with ovarian Mes-HGSC compared to other molecular subtypes.

## Introduction

**FIGURE 1:**
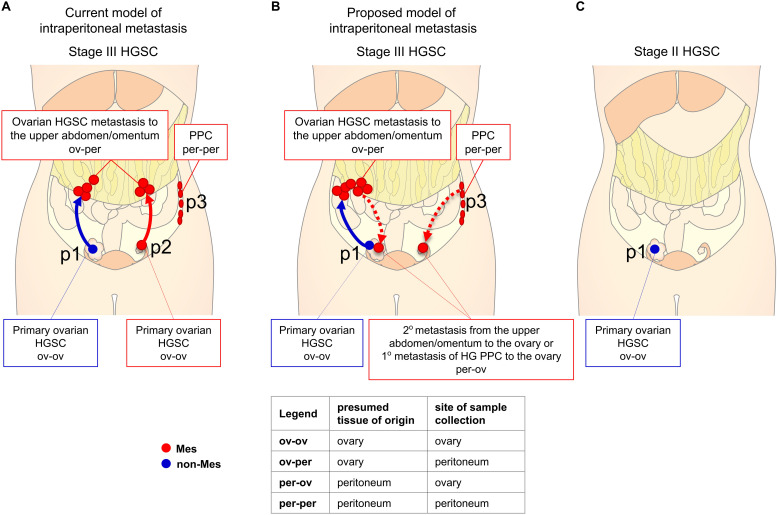
Diagram of peritoneal dissemination of HGSC. For graphical purposes, only stages II and III are shown and up to 3 different primary tumors (p1, p2, p3) occurring in individual patients are shown as if they occurred in a single patient. In stage III HGSC, metastases from the ovary to the upper abdomen/omentum (ov-per) and PPC (per-per) usually exhibit the Mes molecular subtype (red). Primary ovarian HGSC are mostly of the non-Mes subtype (blue) but a subset exhibits the Mes subtype (red). **(A)** In the current model of ovarian cancer dissemination, tumors spread in one direction – from the pelvis to the upper abdomen/omentum (ov-per). Primary ovarian HGSC of the non-Mes and Mes subtype form metastases of the Mes subtype. **(B)** In the proposed model of peritoneal metastasis, tumors spread in both directions – from the pelvis to the upper abdomen/omentum (ov-per) and from the upper abdomen/omentum (HGSC metastases or PPC) to the pelvis (per-ov). True primary ovarian HGSC (ov-ov) are of the non-Mes subtype while metastases from the upper abdomen/omentum to the ovary (per-ov) are of the Mes subtype. **(C)** In stage II HGSC, masses in the ovary are always of the non-Mes subtype because upper abdominal/omental tumors are absent.

High-grade serous carcinoma is the most common and most deadly type of ovarian cancer (Matulonis et al., 2016). The majority of ovarian cancer patients with HGSC are diagnosed with tumors involving one or both ovaries and various additional intraperitoneal sites including the upper abdomen/omentum (FIGO stage III) (Matulonis et al., 2016). HGSC can arise from the fallopian tube, the ovarian surface serous epithelium, or extraovarian peritoneal tissues as primary peritoneal carcinoma (PPC) (Figure 1A). Currently, it is thought that ovarian cancer cells shed from the primary tumor into the peritoneal fluid and disseminate in the peritoneal cavity, typically from the ovary to the upper abdomen/omentum (Figure 1A). However, the model of primarily unidirectional HGSC metastasis from the pelvis to the upper abdomen/omentum seems simplistic within a cavity that lacks internal physical barriers to cancer dissemination. We propose that in stage III HGSC, metastases and PPC in the upper abdomen/omentum shed cancer cell-stroma aggregates into the peritoneum, resulting in intraperitoneal dissemination that includes secondary metastases to the primary tumor in the pelvis (Figure 1B). Patterns of cancer dissemination within the peritoneal cavity have been difficult to discern using genomic data because genomic instability is an early event in HGSC and copy number profiles and mutational patterns are typically shared across different anatomic sites (Brodsky et al., 2014; Lee et al., 2015; Schwarz et al., 2015; Eckert et al., 2016; McPherson et al., 2016). However, individual clones have been tracked using whole-genome and single-nucleus sequencing of patient-matched tumor deposits at different anatomic locations. These studies identified evidence of metastases to the ovary or the fallopian tube in 4 out of 15 patients, thereby demonstrating that re-seeding of the primary tumor site by clones from peritoneal metastases is not a rare event (Eckert et al., 2016; McPherson et al., 2016).

Transcriptomic analyses have clustered primary ovarian HGSC into 4 main molecular subtypes: Immunoreactive, Mesenchymal (Mes), Proliferative and Differentiated (The Cancer Genome Atlas Research Network, 2011; Verhaak et al., 2013). The feature that distinguishes primary ovarian HGSC of the Mes molecular subtype (Mes-HGSC) from the other 3 subtypes (non-Mes subtypes) is the elevated expression of myofibroblast/extracellular matrix (ECM) remodeling genes (Zhang et al., 2015, 2018; Jia et al., 2016). In addition to this distinct transcriptome, primary ovarian Mes-HGSC is more frequently associated with the presence of upper abdominal/omental metastases (Vargas et al., 2015; Torres et al., 2017), suboptimal surgical debulking (presence of residual macroscopic disease after cytoreductive surgery) (Liu et al., 2015; Torres et al., 2017; Wang et al., 2017), severe postoperative complications (Torres et al., 2017, 2018a), and reduced overall survival (Liu et al., 2015; Vargas et al., 2015; Torres et al., 2017, 2018a; Wang et al., 2017; Shilpi et al., 2019) in comparison to the primary ovarian non-Mes subtypes. The current theory suggests that cancer cells in primary ovarian Mes-HGSC recruit myofibroblasts or convert the local ovarian stroma into myofibroblasts, which equip cancer cells with greater metastatic ability (Torres et al., 2017, 2018b; Zhang et al., 2018). However, this theory does not explain why metastases are predominantly of the Mes phenotype even when the primary tumor is Immunoreactive, Proliferative, or Differentiated subtype (Tan et al., 2018). PPC is also typically of the Mes phenotype (Gao et al., 2016), suggesting that this phenotype is an inherent feature of peritoneal lesions.

In this study, we used transcriptomic analyses of tumor samples with annotated presumed sites of tumor origin and sites of sample collection (Supplementary Table S1) to show that the Mes gene signature is expressed in the stromal component of tumors in the upper abdomen/omentum but not in most primary ovarian HGSC. However, if a tumor in the ovary expresses the Mes gene signature, we propose that this tumor contains the microenvironment from tumors in the upper abdomen/omentum. This can occur by two mechanisms (Figure 1B). The first mechanism involves a primary tumor in the ovary/pelvis that metastasizes to the omentum/upper abdomen, then the metastases, which have now acquired the Mes phenotype, continue to seed the rest of the peritoneal cavity, including the ovary where the tumor initially originated. The second mechanism involves PPC from the upper abdomen/omentum that metastasizes throughout the peritoneal cavity, including to the ovary. If our hypothesis is correct, we predict that stage II primary ovarian HGSC (tumors confined to the pelvis) cannot exhibit the Mes subtype because of the absence of cancer/stroma cell aggregates from the upper abdomen/omentum as a source of metastases to the ovary (Figure 1C).

## Results

### The Mes Subtype Reflects Tumor Location

**FIGURE 2:**
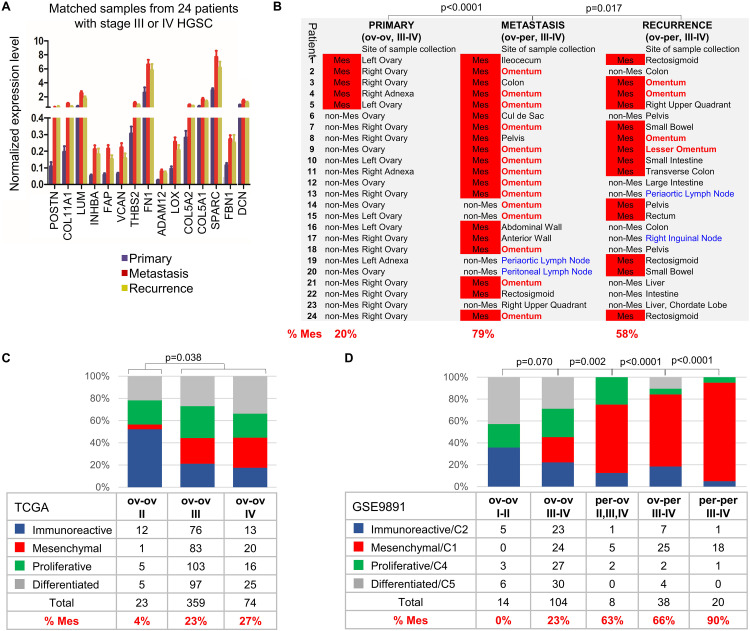
The Mes subtype is characteristic of upper abdominal/omental metastases and PPC while HGSC confined to the pelvis does not exhibit the Mes subtype. **(A)** NanoString expression of 15 mesenchymal genes in samples from matched primary, metastatic, and recurrent stage III-IV HGSC from 24 patients (see “Materials and Methods”). **(B)** The samples were classified into Mes (red) and non-Mes subtypes using the Mes 15-gene z-score. The site of sample collection is indicated for each tumor, with the omentum and lymph nodes indicated in red and blue, respectively. **(C)** Distribution of molecular subtypes by disease stage in the ovarian TCGA data set. Excluded from the analysis were 4 samples for which the ovary was not the presumed site of tumor origin or the site of tumor collection (2 fallopian tube and 2 omentum samples). Additionally, 81 samples that did not cluster among the 4 molecular subtypes were excluded. The *p* value indicates the two-tailed Fisher’s probability test for the number of Mes and non-Mes (Immunoreactive, Proliferative, and Differentiated) samples. **(D)** Distribution of molecular subtypes by disease stage, site of presumed tumor origin, and site of sample collection in the GSE9891 data set. Included in the analysis were only tumors annotated as high grade (2 or 3); serous histology; malignant; stage I, II, III or IV; molecular subtype C1/Mesenchymal, C2/Immune, C5/Differentiated or C4/Proliferative; primary site ovary (ov) or peritoneum (per); and collection site ovary (ov) or peritoneum/colon/omentum (per). Due to the small number of stage I and stage IV samples, stage I and II samples were grouped as stage I-II, and stage III and IV samples were grouped as stage III-IV. One stage II per-per sample was grouped with 7 stage III-IV per-per samples. The p value indicates the two-tailed Fisher’s probability test for the number of Mesenchymal/C1 and non-Mesenchymal (Immunoreactive/C2, Proliferative/C4, and Differentiated/C5) samples.

We examined whether the Mes phenotype varies across patient-matched samples of primary, metastatic, and recurrent HGSC. The Mes phenotype was determined by threshold expression of 15 mesenchymal genes (section “Materials and Methods”; Supplementary Table S2). Notably, these 15 genes were expressed at significantly higher levels in the tumor stroma in comparison to epithelial cancer cells in both the primary HGSC data set of laser-capture-microdissected stromal and epithelial cells (GSE40595 data set) (Yeung et al., 2013; Supplementary Figure S1A) and flow cytometry-isolated individual cells in colorectal cancer (GSE39395 data set) (Calon et al., 2012; Supplementary Figure S1B). The 15 genes were among the top 100 genes used by Verhaak et al. (2013) to define the Mes subtype by expression profile analysis (Mes 100-gene set) (Supplementary Table S2). In the HGSC TCGA data set, this Mes 15-gene signature was associated with poor overall and progression-free survival (Supplementary Figure S1C) and was equivalent to the Mes 100-gene signature in classifying samples of the Mes subtype (Supplementary Figure S2). All 15 genes were expressed at higher levels in the metastatic and recurrent tumors compared to the matched primary tumors (Figure 2A). While only 20% of the primary ovarian HGSC were classified as Mes, 79% of the concurrent and 58% of the recurrent HGSC metastases were classified as Mes (Figure 2B). The higher percentage of the Mes subtype in the concurrent metastases than in the recurrent metastases may be attributed to different sites of sample collection. The omentum was the most common collection site for concurrent metastases but not for recurrent metastases because most patients had a partial omentectomy during primary debulking surgery (Figure 2B). Notably, 15 of 17 metastatic and recurrent samples collected from the omentum were classified as Mes and none of the 4 metastatic and recurrent samples collected from the lymph nodes was classified as Mes (Figure 2B). In 2 patients, omental metastases were classified as non-Mes; their expression levels of the 15 Mes genes were high (Supplementary Table S3) but did not reach the threshold we set for Mes subtype classification (see section “Materials and Methods”).

Since most metastases located in the upper abdomen/omentum were classified as Mes (Figure 2B), we hypothesized that primary ovarian HGSC of the Mes subtype are actually metastases from the upper abdomen/omentum to the ovary. If this is correct, HGSC collected from patients with cancer confined to the pelvis (stage I-II) should not exhibit the Mes phenotype because of the absence of upper abdominal/omental disease as a source of metastatic tumor clusters able to seed the ovary (Figure 1C). We tested this hypothesis in 2 public data sets in which samples had been divided into 4 molecular subtypes. In this study, we used the original molecular classifications for the TCGA and GSE9891 data sets since this classification is most commonly used in the literature (Tothill et al., 2008; The Cancer Genome Atlas Research Network, 2011). Of note, subsequent studies may have used different classification algorithms, which resulted in different molecular subtype assignments to the same samples. In a recent study, 22% of the samples had been reclassified to a different molecular subtype, which resulted in better correlation with survival outcomes (Shilpi et al., 2019).

In the ovarian TCGA data set, samples had been divided into 4 molecular subtypes: Immunoreactive, Mesenchymal (Mes), Proliferative, and Differentiated (The Cancer Genome Atlas Research Network, 2011). According to the strict TCGA guidelines for sample collection, all of the ovarian TCGA tumor samples presumably originated in the ovary and were collected from the ovary (ov-ov) (Figure 2C). Of 23 stage II HGSC samples, only 1 was classified as Mes (Figure 2C). Notably, that sample (TCGA-61-2133) had features of an aggressive malignancy despite its stage II designation: it was annotated as stage IIc, grade 3 with extensive lymphovascular permeation, positive pelvic lymph nodes and the shortest overall survival among patients with stage IIc HGSC who died from the disease (676 days vs 1,380 days mean survival). In contrast to stage II HGSC, out of 359 stage III HGSC and 74 stage IV HGSC, Mes tumors contributed to 23 and 27% of samples, respectively (Figure 2C).

In the GSE9891 data set, HGSC samples had been clustered into C1/Mesenchymal, C2/Immunoreactive, C4/Proliferative, and C5/Differentiated molecular subtypes (Tothill et al., 2008) and annotated by their presumed tissue of origin and the site of specimen collection as ov-ov, per-ov, ov-per, and per-per (Figure 2D). Due to a small number of stage I and stage IV samples, we grouped stage I and II samples as stage I-II, and stage III and IV samples as stage III-IV. None of the 14 ov-ov stage I-II HGSC was classified as Mes/C1 (Figure 2D). Of the 104 ov-ov stage III-IV HGSC, 23% were classified as C1/Mes. Of the 38 ov-per stage III-IV HGSC, 66% were classified as C1/Mes (Figure 2D). Of the 8 per-ov HGSC (including 1 stage II and 7 stage III-IV), 63% were classified as C1/Mes (Figure 2D). According to our hypothesis, all metastases that originated in the upper abdomen/omentum as PPC and then spread to the ovary (per-ov) should be of the C1/Mes subtype. However, it is important to note that these 8 per-ov tumors were reported by pathologists as PPC based only on the impression of disease distribution gathered from the surgeon’s description of the intraoperative findings in the operative report and the tissue samples that surgeons had elected to excise. Interestingly, and as noted by pathologists in some of these surgical pathology reports, pathologists were not always certain or in agreement about the origin of the tumor (primary ovarian vs. PPC). Of the 20 per-per stage III-IV HGSCs, 90% were classified as C1/Mes. It is unknown if some of these PPC samples were located in the pelvis, in which case we predict the non-Mes phenotype. As an independent method of classification, we used the Mes 15-gene signature (Supplementary Table S2) to classify samples in the TCGA (Figure 2C) and GSE9891 (Figure 2D) data sets into Mes and non-Mes subtypes. This classification resulted in similar proportions of Mes samples in each respective group (Supplementary Tables S4, S5 and Figure S3) as the original classification of the molecular subtypes in Figures 2C,D.

Together, we conclude that ov-ov HGSC stage I-II (confined to the pelvis) are almost never of the Mes subtype while ∼20–30% of ov-ov HGSC stage III-IV (presence of concurrent upper abdominal/omental metastases) are of the Mes subtype. HGSC samples collected from the peritoneal cavity (ov-per and per-per) as well as samples presumed to be peritoneal metastases to the ovary (per-ov) are typically of the Mes subtype.

### The Mes Molecular Subtype Is Defined by the Metastatic Microenvironment, Not the Epithelial Cancer Cells

**FIGURE 3:**
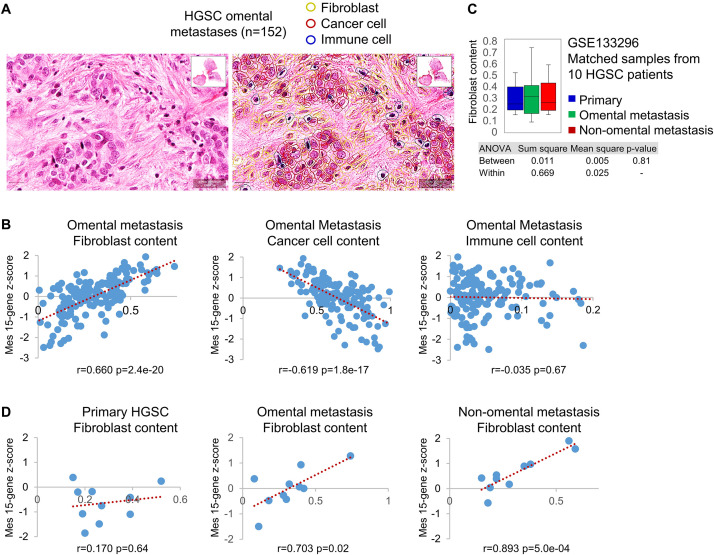
The Mes gene signature expression correlates with fibroblast content in HGSC metastases but not primary ovarian HGSC. **(A)** A representative example of cell type (fibroblast, epithelial cancer cell, immune cell) annotation by QuPath analysis of H&E-stained full sections of omental metastases isolated from 152 HGSC patients at the time of primary debulking surgery (GSE135712). **(B)** Correlation of the Mes 15-gene z-score (Y axis) with the content of fibroblasts, cancer cells, and immune cells in omental metastases isolated from 152 HGSC patients (GSE135712). The content of each cell type was determined as the percent of one cell type in the 3 annotated cell types (fibroblasts, cancer cells, immune cells) in each sample (X axis). **(C)** Fibroblast content in matched primary tumors, omental metastases, and non-omental peritoneal metastases isolated at the time of primary debulking surgery from 10 HGSC patients (GSE133296). Fibroblast content was determined as the percent of fibroblasts in the 3 annotated cell types in each sample. **(D)** Correlation of the Mes 15-gene z-score (Y axis) with the content of fibroblasts (X axis) individually in matched primary tumors, omental metastases, and non-omental intraperitoneal metastases from 10 HGSC patients (GSE133296).

To determine which cell type expresses the Mes 15-gene signature, we used digital image analysis for the annotation of fibroblasts, epithelial cancer cells, and immune cells in H&E-stained full sections of omental metastases collected during primary debulking surgery from 152 HGSC patients (GSE135712) (Figure 3A). The Mes 15-gene z-score was determined for each patient (Supplementary Figure S4A) and correlated with the content of each of the 3 annotated cell types. The two prevalent cell types in omental metastases were epithelial cancer cells and fibroblasts, while the content of immune cells was variable across 152 samples (data not shown). The Mes 15-gene z-score correlated with the fibroblast content (*r* = 0.660; *p* = 2.4e-20), inversely correlated with the epithelial cancer cell content (*r* = -0.619; *p* = 1.8e-17) and showed no significant correlation with the immune cell content (*r* = -0.035; *p* = 0.67) (Figure 3B), suggesting that among these 3 cell types in omental metastases, fibroblasts are the most likely source of the Mes 15-gene signature.

To study the correlation of the Mes 15-gene z-score with fibroblast content in primary and metastatic tumors, we used concurrent primary ovarian HGSC, omental metastases, and non-omental intraperitoneal metastases collected at the time of primary debulking surgery from 10 HGSC patients (GSE133296). The average fibroblast content did not differ significantly between primary ovarian HGSC, omental metastases, and non-omental metastases (Figure 3C). The Mes 15-gene z-score (Supplementary Figure S4B) was significantly correlated with the fibroblast content in omental (*r* = 0.703; *p* = 0.02) and non-omental intraperitoneal (*r* = 0.893; *p* = 5.0e-4) metastases but not in primary tumors (*r* = 0.170; *p* = 0.64) (Figure 3D), suggesting that primary tumor fibroblasts were not expressing high levels of the 15 Mes genes. This result is consistent with our prior *in situ* hybridization findings that only a small number of patients (∼20%) expressed COL11A1 in cancer-associated fibroblasts (CAFs) in primary ovarian HGSC, while the majority of patient-matched metastases expressed COL11A1 in CAFs (Cheon et al., 2014).

The Mes 15-gene z-score was inversely correlated with the content of epithelial cancer cells and was not significantly different between primary ovarian HGSC, omental metastases, and non-omental metastases (data not shown). The Mes 15-gene z-score was not enriched in metastases if metastatic epithelial cancer cells were stripped of their microenvironment. EpCAM-positive epithelial cancer cells isolated from matched primary ovarian HGSC, ascites, and metastasis from 5 patients (3 with replicate samples) (GSE73168) (Gao et al., 2019) exhibited equivalent relative values of the Mes 15-gene z-score (Supplementary Figures S4C,D). Together, these results show that the Mes phenotype is determined by the metastatic microenvironment rather than by the intrinsic molecular subtype of epithelial cancer cells.

### Primary Ovarian HGSC of the Mes Subtype Are Enriched for a Gene Signature Characteristic of Stromal Cells in Metastases Located in the Upper Abdomen/Omentum

**FIGURE 4:**
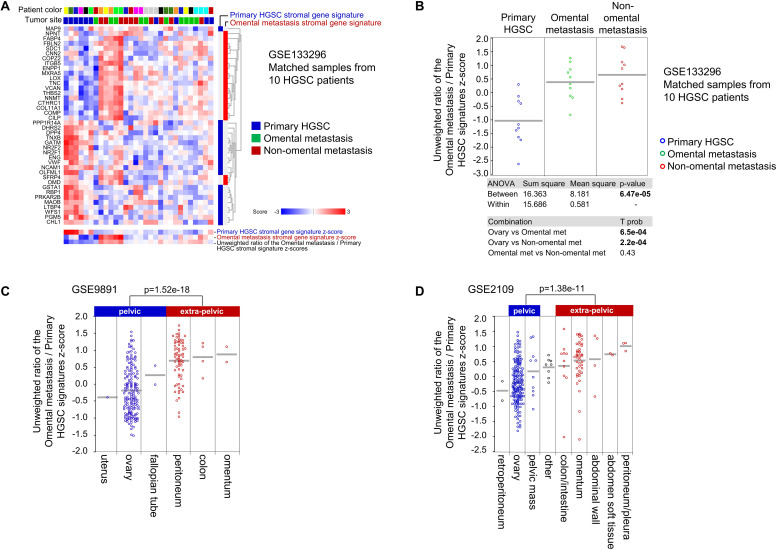
The stroma in HGSC metastases has different molecular features than the stroma in primary ovarian HGSC. **(A)** Euclidean clustering heatmap of expression values of 2 public stromal gene signatures (Primary HGSC stromal gene signature and Omental metastasis stromal gene signature derived from laser-capture-microdissected stromal cells in matched primary ovarian HGSC and mental metastases from 11 patients with HGSC) applied to the GSE133296 transcriptome data set of matched primary ovarian HGSC, omental metastases, and non-omental metastases from 10 HGSC patients. Blue and red bars on the right indicate which genes belong to the primary ovarian HGSC stromal gene signature (blue) and the omental metastasis stromal gene signature (red). Transcripts for GSTA2 (from the original primary ovarian HGSC stromal gene signature) and LPREL2 (from the original omental metastasis stromal gene signature) were missing in the GSE133296 data set. The gene signature z-score was defined as the average z-score of a z-score-transformed GSE133296 data set. The average gene signature z-scores and the average unweighted ratio of the gene signature z-scores are shown at the bottom of the heatmap. **(B–D)** Dot plots of the ratio of z-scores from the Omental metastasis stromal gene signature (positive unweighted value) and Primary HGSC stromal gene signature (negative unweighted value) in panel **(B)** primary ovarian HGSC, omental metastases, and non-omental metastases in the GSE133296 data set; **(C)** different sites of sample collection in the GSE9891 data set (excluded from the analysis were tumors of low malignant potential, non-serous tumors, one bone metastasis, and tumors lacking annotation of the collection site); and **(D)** different sites of sample collection in the GSE2109 data set (included in the analysis were ovarian tumors of all types and histologies that have been annotated by the site of sample collection; some sites have been grouped in this graph; for original annotation, please see Supplementary Table S6). The GSTA2 transcript was missing in the GSE9891 and GSE2109 data sets. The bars indicate average ratio of z-scores in each group.

We were interested to know if the stroma in primary ovarian HGSC differs from the stroma in HGSC metastases located in various tissue sites in the peritoneal cavity. To completely exclude the epithelial cancer cell transcriptome from the analysis, we used published stromal gene signatures derived from proteome data of laser-capture-microdissected stromal cells from primary ovarian HGSC and matched omental metastases from 11 HGSC patients (Eckert et al., 2019). We first validated the 2 stromal gene signatures (Eckert et al., 2019) in our own data set (GSE133296) of matched primary ovarian HGSC, omental metastases, and non-omental metastases from 10 HGSC patients. The primary ovarian HGSC stromal gene signature was overexpressed in a subset of primary ovarian HGSC while the omental metastasis stromal gene signature was overexpressed in a subset of omental and non-omental metastases (Figure 4A). Thus, application of the published proteome-derived stromal gene signatures to our data set shows that the 2 stromal gene signatures are differentially enriched in primary and metastatic tumors in most patients (Figure 4A). To assign a quantitative value to the difference in enrichment of the 2 stromal gene signatures, we used an unweighted ratio of the stromal gene signature z-scores (positive value for the omental metastasis gene signature and negative value for the primary ovarian HGSC gene signature). The average unweighted ratio of the z-scores from Omental metastasis/Primary HGSC stromal gene signatures was significantly lower in primary ovarian HGSC compared to patient-matched omental or non-omental metastases in the GSE133296 data set (Figure 4B).

Using 2 large transcriptomic data sets in which subsets of samples have been annotated by the site of sample collection (GSE9891 and GSE2109), we showed that the average unweighted ratio of the z-scores from Omental metastasis/Primary HGSC stromal gene signatures was lower in tumors located retroperitoneally or in the pelvis (ovary, uterus, and fallopian tube) than in tumors located outside of the pelvis (omentum, colon/intestine, abdominal wall, peritoneum, and diaphragm) (Figures 4C,D). Together, these data suggest that intraperitoneal tumors located in the pelvis are enriched for a stromal gene signature of primary ovarian HGSC while tumors outside of the pelvis are enriched for a stromal gene signature of omental metastases.

**FIGURE 5:**
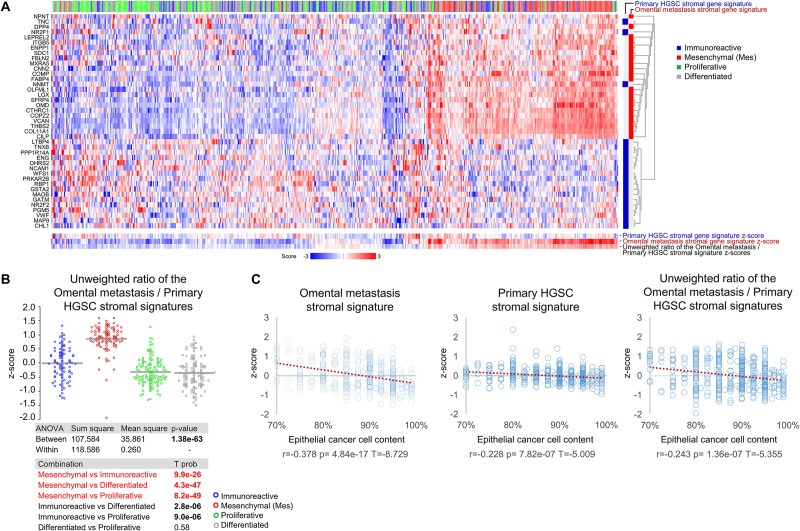
TCGA primary ovarian Mes-HGSC are enriched for a stromal gene signature of omental metastases. **(A)** Euclidean clustering heatmap of expression values of 2 public stromal gene signatures (Primary HGSC stromal gene signature and Omental metastasis stromal gene signature derived from laser-capture-microdissected stromal cells in matched primary ovarian HGSC and omental metastases from 11 patients with HGSC) applied to the TCGA primary ovarian HGSC samples classified as the Immunoreactive, Mesenchymal, Proliferative and Differentiated molecular subtypes (excluded from the analysis were 4 samples that were not collected from the ovary and 81 samples that did not cluster among the 4 molecular subtypes). Blue and red bars on the right indicate which genes belong to the primary ovarian HGSC stromal gene signature (blue) and the omental metastasis stromal gene signature (red). The primary ovarian HGSC stromal gene signature was represented by 20 of the original 21 genes genes (GSTA2 transcript was missing in the TCGA data set). The gene signature z-score was defined as the average z-score of a z-score-transformed TCGA data set. The average gene signature z-scores and the average unweighted ratio of gene signature z-scores are shown at the bottom of the heatmap. **(B)** Dot plot of the ratio of z-scores from the Omental metastasis stromal gene signature (positive unweighted value) and Primary HGSC stromal gene signature (negative unweighted value) in the Immunoreactive, Mesenchymal, Proliferative and Differentiated molecular subtypes in the TCGA data set. **(C)** Dot plots of Spearman correlation of stromal gene signatures z-scores and percent of epithelial cancer cells present in histological sections of tumor samples in the TCGA data set.

To determine whether the Omental metastasis and Primary HGSC stromal gene signatures are associated with molecular subtypes in primary ovarian HGSC, we used the ovarian TCGA data set (The Cancer Genome Atlas Research Network, 2011). Overlay of the Omental metastasis and Primary HGSC stromal gene signatures with the ovarian TCGA data set showed strong enrichment of the Omental metastasis gene signature in the Mes subtype while the Primary HGSC stromal gene signature was not significantly enriched in any specific molecular subtype (Figure 5A). The average unweighted ratio of the z-scores from Omental metastasis/Primary HGSC stromal gene signatures was significantly enriched in the Mes subtype in comparison to the Immunoreactive, Differentiated, and Proliferative molecular subtypes (Figure 5B). Since the Immunoreactive and Mesenchymal primary ovarian HGSC subtypes have been shown to contain more stroma (less epithelial cancer cells) than the Differentiated and Proliferative subtypes (Aran et al., 2015; Zhang et al., 2015, 2018; Chen et al., 2018), we considered the possibility that the stromal gene signatures are overexpressed in samples with high stromal content and underexpressed in samples with high epithelial cancer cell content. However, we show little correlation between the stromal gene signatures and epithelial cancer cell content in the TCGA data set (Figure 5C), suggesting that the strong enrichment of the average unweighted ratio of the z-scores from Omental metastasis/Primary HGSC stromal gene signatures in Mes-HGSC (Figure 5B) reflects a molecularly different type of stroma rather than an increased presence of the stroma in the Mes subtype. Together, these results indicate that the Mes-HGSC subtype is enriched for cells that exhibit the phenotype of stromal cells in omental metastases rather than stromal cells in primary ovarian HGSC.

## Discussion

Molecular profiling studies have identified 4 distinct molecular subtypes of primary ovarian HGSC of which the Mes subtype has the lowest rate of optimal surgical debulking and the worst overall survival (Tothill et al., 2008; Tan et al., 2013; Verhaak et al., 2013; Konecny et al., 2014; Zhang et al., 2015, 2016; Torres et al., 2017; Wang et al., 2017), and is almost always associated with coexisting upper abdominal/omental metastases (Torres et al., 2017; Wang et al., 2017). It has been shown that cancer-associated stroma and ECM largely contribute to the Mes gene signature (Zhang et al., 2015, 2018; Jia et al., 2016). Additionally, it has been shown that the transcriptome of primary ovarian Mes-HGSC is strongly correlated with the stromal gene signature of omental metastases (Eckert et al., 2019). Considering the phenotypic similarity between primary ovarian Mes-HGSC and peritoneal metastases and the frequent coexistence of primary ovarian Mes-HGSC with upper abdominal/omental metastases, we propose that primary ovarian Mes-HGSC might actually be cancer-stroma aggregates that detached from tumors located in the upper abdomen/omentum. Indeed, whole-genome and single-nucleus sequencing analyses have demonstrated that metastases are not always unidirectional and that the re-seeding of peritoneal metastasis to the fallopian tube or ovary can occur (Eckert et al., 2016; McPherson et al., 2016). Unfortunately, the gene expression based molecular subtypes of such samples cannot be determined as these studies isolated high purity epithelial tumor cells rather than stromal cells, which frequently contribute to gene expression signatures that define molecular subtypes.

The signature genes in the Mes molecular subtype are frequently referred to in literature as the signature genes of epithelial-mesenchymal transition (EMT) because the same genes can be upregulated in epithelial cells in cell culture upon induction of EMT, i.e., by knockout of E-cadherin (Onder et al., 2008), overexpression of Twist (Yang et al., 2004), and exposure of epithelial cancer cells to TGFβ or conditioned media from cancer-associated fibroblasts (Beach et al., 2016). We have shown that laser-capture-microdissected epithelial cells from primary HGSC express negligible levels of the 15 Mes genes in comparison to laser-capture-microdissected stromal cells. Thus, even if epithelial cancer cells undergo partial EMT, the levels of mesenchymal genes expressed in cancer cells would be dwarfed by the levels of mesenchymal genes expressed in fibroblasts in mixed tumor samples. It has been suggested that one possible source of CAFs are epithelial cancer cells that undergo full EMT and thus are indistinguishable from fibroblasts (Petersen et al., 2003). However, studies in different solid tumors, including breast and ovarian carcinomas, have shown that CAFs typically do not contain genetic mutations present in epithelial cancer cells, suggesting that CAFs do not originate from cancer cells (Qiu et al., 2008; Hosein et al., 2010). While it is possible that a minor fraction of CAFs in the mesenchymal subtype of ovarian cancer arises through EMT, this fraction of CAFs is probably below the detection levels of RNA profiling technologies employed in the data sets (Supplementary Table S1) used in our analyses.

Studies in cell co-cultures and in mouse models demonstrated the existence of heterotypic aggregates of cancer cells and stroma, in which stromal cells support epithelial cancer cell survival and guide peritoneal invasion, and can accompany epithelial cancer cells to a new metastatic site and actively reconstitute the tumor stroma in newly formed metastases (Duda et al., 2010; Gao et al., 2019). However, it has been shown that HGSC metastases rarely contain CAFs from primary ovarian HGSC (Gao et al., 2019), suggesting that stromal cells in primary ovarian tumors are not overly efficient in accompanying cancer cells to a new metastatic site and/or are not proficient in re-building the stroma at a new site. It is likely that implantation of cancer cell-stroma aggregates at a new metastatic site requires significant remodeling of the local stroma or recruitment of new stroma. Indeed, stroma in the upper abdominal/omental metastases is frequently enriched for markers of myofibroblasts and ECM remodeling, such as POSTN, COL11A1, LOX, VCAN, TNC, and THBS2 (Gao et al., 2016; Jia et al., 2016; Eckert et al., 2019). It is possible that the upper abdominal/omental metastasis stroma is more efficient than primary ovarian cancer stroma in accompanying metastatic cancer cells and reconstituting the stroma at secondary metastatic sites. For example, omental adipocytes have been shown to promote ovarian cancer metastasis and provide energy for rapid tumor growth (Nieman et al., 2011, 2013). Presently, it would be difficult to design an experimental system for lineage tracing of CAFs to prove our hypothesis in a mouse model. The existing genetically engineered mouse models that develop upper abdominal metastases from the fallopian tube/ovarian lesions are triple or quadruple transgenics; it is not feasible to combine them with additional lines of transgenic mice for lineage tracing. Additionally, since autochtonous ovarian cancer models have been generated relatively recently, it is unknown if the patterns of ovarian cancer dissemination in the mouse peritoneal cavity recapitulate the patterns of intraperitoneal dissemination in women with ovarian cancer.

Our result that the majority of patient-matched metastatic or recurrent HGSC samples were classified as Mes-HGSC irrespective of the primary cancer subtype is consistent with the results of a recent study in a different cohort of patients (Tan et al., 2018) as well as a study showing that the majority of PPC are classified as the Mes subtype (Gao et al., 2016). According to our hypothesis that the Mes gene signature is a signature of stromal cells in the upper abdominal/omental HGSC, all HGSC in the upper abdomen/omentum should be classified as Mes. Yet in our study, only 90% of PPC and 66% of peritoneal metastases were classified as Mes. It is also expected that all PPC metastases to the ovary are Mes but only 63% were classified as Mes in our study. Multiple technical reasons could explain why some metastases to the ovary did not classify as Mes including imperfections in algorithms that had been used for the Mes subtype classification in the original publications, unknown precise site of sample collection in the peritoneal cavity (pelvis vs upper abdomen/omentum), and/or inclusion of samples that had been annotated as PPC based on the tumor distribution but are actually primary ovarian or fallopian tube HGSC. A biological explanation for the existence of non-Mes metastases in the upper abdomen/omentum could be that metastases are initially associated with accompanying stroma from the primary ovarian HGSC until cancer cells can recruit and/or remodel the stroma at the metastatic site.

Similar to HGSC, colorectal cancer (CRC) has been classified into 4 molecular subtypes (CMS1-4) (Chen et al., 2018). The (Mes) molecular subtype (CMS4) is associated with poor survival (Calon et al., 2015; Guinney et al., 2015). It has been shown that the CMS4 subtype is largely defined by genes expressed in CAFs (Guinney et al., 2015; Dunne et al., 2016; Ubink et al., 2018), challenging the assumption that poor prognosis tumors acquire stem-like characteristics by undergoing widespread EMT. The rationale for classifying tumors by molecular subtypes has been challenged by a study demonstrating that CRC from an individual patient can be simultaneously classified into multiple molecular subtypes based purely on the degree of stromal infiltration in the tumoral region (Dunne et al., 2016). The observed intratumoral heterogeneity was confirmed in the study by Ubink et al. where the CMS4 subtype was defined by qRT-PCR expression of 4 mesenchymal markers (PDGFRA, PDGFRB, PDGFC, and KIT) (Ubink et al., 2017). The issue of intratumoral heterogeneity was somewhat clarified by another study by Ubink et al. in which primary CRC were compared with concurrent peritoneal metastases. In that study, 60% of primary CRC (a higher proportion than in the original CMS classification study (Guinney et al., 2015) and 75% of peritoneal metastases were classified as CMS4. The observed high proportion of the CMS4 subtype in both primary and metastatic CRC is consistent with our data showing that the Mes subtype is determined by the tumor location, which is primarily in the upper abdomen/omentum for both primary and metastatic CRC. Due to differences in the genes used to classify the Mes subtype, we could not directly compare HGSC and CRC classification, however, it is worth noting that expression levels of the Mes 15-gene z-score was higher in primary CRC than in primary HGSC in the TCGA data set (data not shown). Similar to our results in HGSC, CMS4 status of matched primary and metastatic CRC did not correlate with the percentage of stromal cells in the sample, suggesting that the CMS4 phenotype was determined by the functional differences in the type of stroma (Ubink et al., 2017).

Major limitations of our study are the correlative nature of the data and a small number of samples in some of the data sets used for the analyses. Thus, we cannot completely exclude the possibility that cancer cells in primary ovarian Mes-HGSC are capable of converting the resident ovarian stromal cells into myofibroblasts or recruiting myofibroblast-like stroma to the ovary. However, if this were true, it would be expected that some of the stage I-II primary ovarian HGSC were of the Mes subtype. Of the 37 stage I-II primary ovarian HGSC samples that satisfied our inclusion criteria in the TCGA and GSE9891 data sets, only 1 was classified as Mes and that tumor exhibited features of a highly aggressive malignancy (stage IIc with lympho-vascular invasion and early death from the disease), suggesting the potential presence of malignant ascites containing microscopic cancer cell-stroma aggregates from the upper abdomen/omentum.

Although the main purpose of this study was to present a new perspective in the understanding of intraperitoneal HGSC dissemination, our results have clinical relevance. We suggest that the Mes gene signature in primary ovarian HGSC signifies advanced/high-stage intraperitoneal metastatic dissemination that includes metastasis to the ovary by cancer cell-stroma aggregates from the upper abdomen/omentum. From this perspective, stage III Mes-HGSC could be considered “more advanced” than stage III non-Mes-HGSC. Additionally, our results may be relevant to the future clinical use of molecular subtype biomarkers to triage patients to primary cytoreductive surgery or neoadjuvant chemotherapy. Genes associated with the Mes subtype have been associated with suboptimal debulking and increased postoperative morbidity and mortality (Riester et al., 2014; Tucker et al., 2014; Torres et al., 2017; Wang et al., 2017), suggesting that the Mes subtype could be helpful as a biomarker to triage patients toward neoadjuvant chemotherapy (Orsulic and Karlan, 2019). Some medical centers are already using preoperative biopsy to assess resectability and triage patients for neoadjuvant chemotherapy (Spencer et al., 2010; Nick et al., 2015). Results of the current analysis show that the site of tumor biopsy is important in determining the Mes subtype. Although large omental metastases are most easily accessed (Spencer et al., 2010), they are not reliable for patient stratification by tumor molecular subtype classification because they usually exhibit the Mes subtype. If classification by molecular subtype is to be used to inform clinical management, our findings underscore that biopsies submitted for molecular analysis should be obtained from the ovarian mass, even though it may be more difficult to obtain than an omental biopsy (Nagamine et al., 2017; Rutten et al., 2017).

## Materials and Methods

### Patient Samples and Gene Expression Analyses

Formalin-fixed paraffin-embedded (FFPE) blocks were retrieved from the pathology archives at Cedars-Sinai Medical Center under an approved IRB protocol. FFPE blocks were sectioned onto uncharged glass slides. One 4 μm H&E-stained section was used by a pathologist to circle the tumor areas and delineate them from the adjacent normal tissue. Depending on the tumor size, 1–3 unstained 10 μm sections were macrodissected (removal of non-tumor areas based on the H&E template) with a clean razor blade. Total RNA was isolated using the miRNeasy FFPE kit according to the manufacturer’s instructions (Qiagen). For the GSE135712 data set, samples of omental metastases collected from 152 HGSC patients at the time of primary debulking surgery were analyzed for RNA expression of 1,067 genes by NanoString nCounter technology (NanoString Technologies). Data were normalized using nSolver software (NanoString Technologies). In a separate NanoString data set, matched primary, metastatic, and recurrent HGSC samples from 29 patients were analyzed for RNA expression of 15 genes by NanoString nCounter. Five patients were excluded from the analysis due to missing tissue or missing mRNA data for one of the matched tumors. In 4 patients where more than one matched metastatic or recurrent tumor sample was available, one sample was randomly selected for the study. For the GSE133296 data set, matched HGSC samples collected from the ovary, omental metastasis, and non-omental intraperitoneal metastasis from 10 patients at the time of primary debulking surgery were analyzed for RNA expression by RNA sequencing using the SMARTer Stranded Total RNA-Seq Kit v2 on the Illumina HiSeqX platform (MedGenome). Unwanted sequences (non-polyA tailed RNAs from the sample, mitochondrial genome sequences, ribosomal RNAs, transfer RNAs, adapter sequences and others) were removed using Bowtie2 (version 2.2.4). The paired-end reads were aligned to the reference human genome downloaded from the UCSC database (GRCh37/hg19). STAR (2.4.1) aligner was used for read alignment. Reads mapping to ribosomal and mitochondrial genomes were removed before alignment was performed. The raw read counts were estimated using HTSeq-0.6.1. Read count data were normalized using DESeq2.

### Expression Data Sets

For the ovarian TCGA data set, level 3 data (gene merged) on the AgilentG4502A_07_3 platform was used for analyses. The GSE2109, GSE9891, GSE39395, GSE40595, and GSE73168 data sets were obtained from the Gene Expression Omnibus (GEO) repository. Raw and normalized data for GSE135712 and GSE133296 were deposited into the GEO archive. Data sets used in this study and their associated publications are listed in Supplementary Table S1.

### Gene Signatures Distinguishing Mes-HGSC From Non-Mes-HGSC

For the Mes 15-gene signature, matched primary, metastatic, and recurrent FFPE tumor samples from 24 patients with HGSC were profiled with NanoString nCounter for expression of 15 genes (Supplementary Table S2) that we previously found to be associated with poor survival in HGSC (Cheon et al., 2014) and/or belonged to the pan-cancer gene signature of activated CAFs (Jia et al., 2016). A threshold for each of the 15 genes was determined by its median expression level in primary tumors in the data set of 24 patients with paired primary, metastatic and recurrent tumors. For TCGA and GSE9891 data sets, the threshold for each of the 15 genes was determined by median expression levels in all tumors in each corresponding date set. A score of 1 was given if the expression exceeded the threshold, otherwise a score of 0 was given (Supplementary Tables S3–S5). Once 15 individual scores corresponding to 15 mesenchymal genes were obtained, they were used to create a Mes score. The Mes score was normalized to a range between 0 and 1, in which 1 indicated Mes-HGSC while all other values indicated non-Mes-HGSC. To test the concordance of the Mes 15-gene signature and the original classification of the Mes molecular subtype in the TCGA and GSE9891 data sets, we calculated Cohen’s kappa coefficient between the two classifications. Cohen’s kappa coefficient for Mes and non-Mes classifications in the TCGA data set was 0.733. Application of this 15-gene score algorithm to the TCGA data set correctly classified 95 of 104 (91%) samples annotated as the Mes subtype and 314 of 352 (89%) samples annotated as the non-Mes subtype (Immunoreactive, Proliferative or Differentiated) (Supplementary Table S4). Cohen’s kappa coefficient for Mes and non-Mes classifications in the GSE9891 data set was 0.792. Application of this 15-gene score algorithm to the GSE9891 data set correctly classified 71 of 72 (99%) samples annotated as the Mes/C1 subtype and 94 of 112 (84%) samples annotated as the non-Mes subtype (Supplementary Table S5). For the 100-gene set mesenchymal HGSC gene signature, we used the top 100 genes that distinguished the Mes subtype from other subtypes, according to the study by Verhaak et al. (2013; Supplementary Table S2). The 21-gene stromal signature of primary ovarian HGSC and the 21-gene stromal signature of omental metastasis have been described (Eckert et al., 2019).

### Data Analyses

The R2: Genomic Analysis and Visualization Platform^1^ was used for analyses of RNA expression levels and correlation between gene signatures and sample groups in different data sets. The gene signature z-score was defined as the average z-score of a z-score-transformed data set. For digital image data analyses, H&E stained slides were scanned at 20× magnification using Aperio AT Turbo. The image analysis was performed using the QuPath software. The image analysis workflow consisted of cell/nucleus detection, annotation of regions containing 3 different cell types (fibroblast, epithelial cancer cell, immune cell), creating the cell detection classifier, and applying the classifier to all cells in the circled regions of the slide.

## Data Availability Statement

The datasets presented in this study can be found in online repositories. The names of the repository/repositories and accession number(s) can be found in the article/Supplementary Material.

## Ethics Statement

The studies involving human tissues were reviewed and approved by Cedars-Sinai Medical Center IRB Committee.

## Author Contributions

SO conceived the hypothesis, analyzed data sets and wrote the manuscript. JL reviewed and categorized clinical information. AW reviewed the pathology and selected tissue blocks for mRNA isolation. BT-H isolated RNA from FFPE samples and conducted NanoString nCounter analyses. YH, YR, MH, MR, and ET contributed to data analyses. JM analyzed the NanoString nCounter data for matched primary, metastatic, and recurrent tumor samples. AW and BK provided critical input and contributed to the writing of the manuscript. All authors participated in manuscript revisions.

## Conflict of Interest

SO and BK have a patent “Molecular Signatures of Ovarian Cancer (US010253368 and EU2908913)”. The remaining authors declare that the research was conducted in the absence of any commercial or financial relationships that could be construed as a potential conflict of interest. The handling Editor declared a shared affiliation with several of the authors YH, BT-H, YR, MH, MR, ET, JM, BY, and SO at time of review.

## Footnotes

1 http://hgserver1.amc.nl/cgi-bin/r2/main.cgi
