# Supplementary material for: Are Epithelial Ovarian Cancers of the Mesenchymal Subtype Actually Intraperitoneal Metastases to the Ovary?
Source: Front Cell Dev Biol. 2020 Jul 17;8:647. doi: 10.3389/fcell.2020.00647 (PMC7380132; doi:10.3389/fcell.2020.00647)
Supplement: Supplementary file 1 [file Data_Sheet_1.PDF]

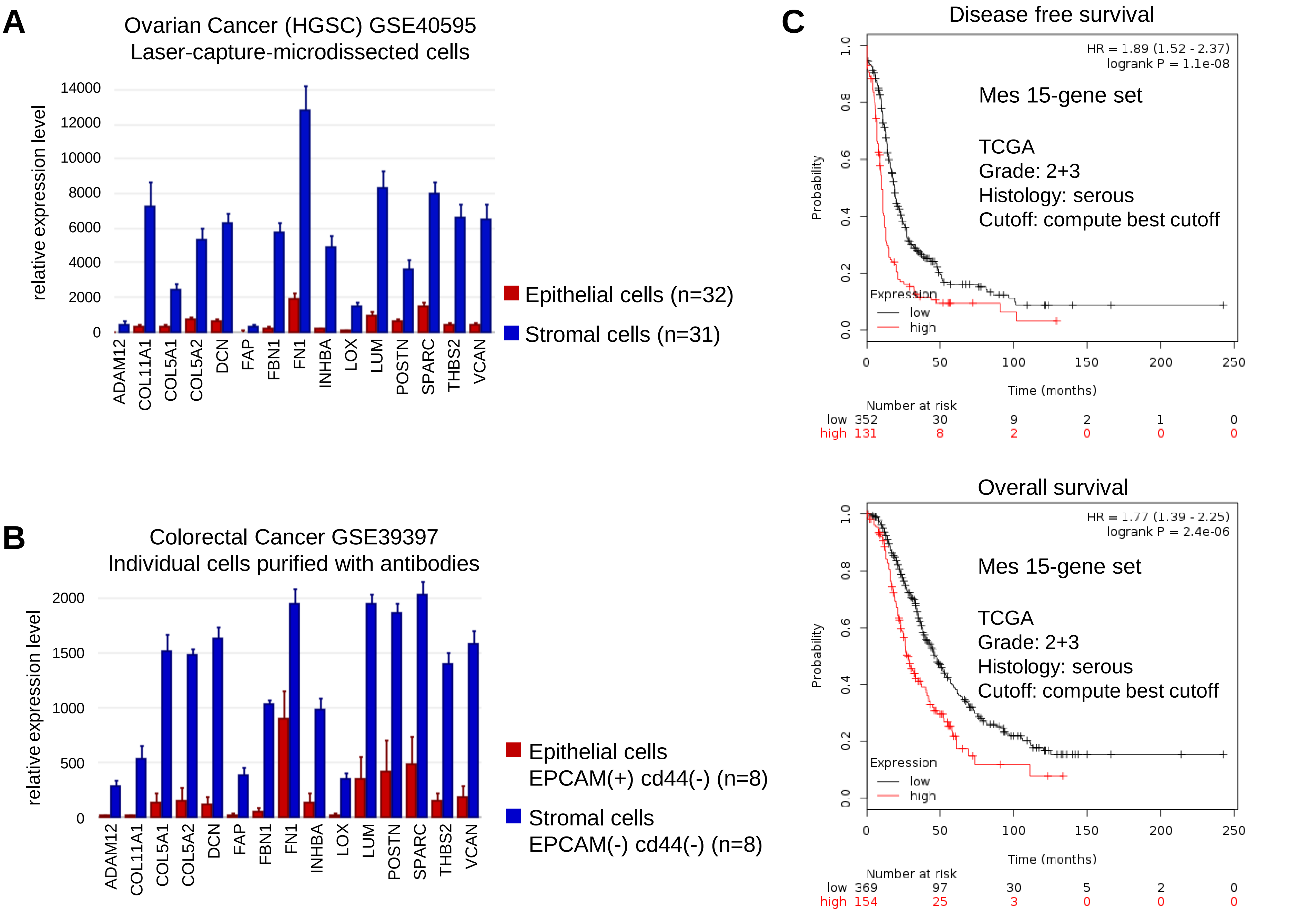
A
Ovarian Cancer (HGSC) GSE40595
Laser-capture-microdissected cells
relative expression level
14000
12000
10000
8000
6000
4000
2000
0
ADAM12
COL11A1
COL5A1
COL5A2
DCN
FAP
FBN1
FN1
INHBA
LOX
LUM
POSTN
SPARC
THBS2
VCAN
Epithelial cells (n=32)
Stromal cells (n=31)
B
Colorectal Cancer GSE39397
Individual cells purified with antibodies
relative expression level
2000
1500
1000
500
0
ADAM12
COL11A1
COL5A1
COL5A2
DCN
FAP
FBN1
FN1
INHBA
LOX
LUM
POSTN
SPARC
THBS2
VCAN
Epithelial cells
EPCAM(+) cd44(-) (n=8)
Stromal cells
EPCAM(-) cd44(-) (n=8)
C
Disease free survival
HR = 1.89 (1.52 - 2.37)
logrank P = 1.1e-08
Mes 15-gene set
TCGA
Grade: 2+3
Histology: serous
Cutoff: compute best cutoff
Probability
Expression
low
high
Time (months)
Number at risk
low 352 30 9 2 1 0
high 131 8 2 0 0 0
Overall survival
HR = 1.77 (1.39 - 2.25)
logrank P = 2.4e-06
Mes 15-gene set
TCGA
Grade: 2+3
Histology: serous
Cutoff: compute best cutoff
Probability
Expression
low
high
Time (months)
Number at risk
low 369 97 30 5 2 0
high 154 25 3 0 0 0

Fig. S1

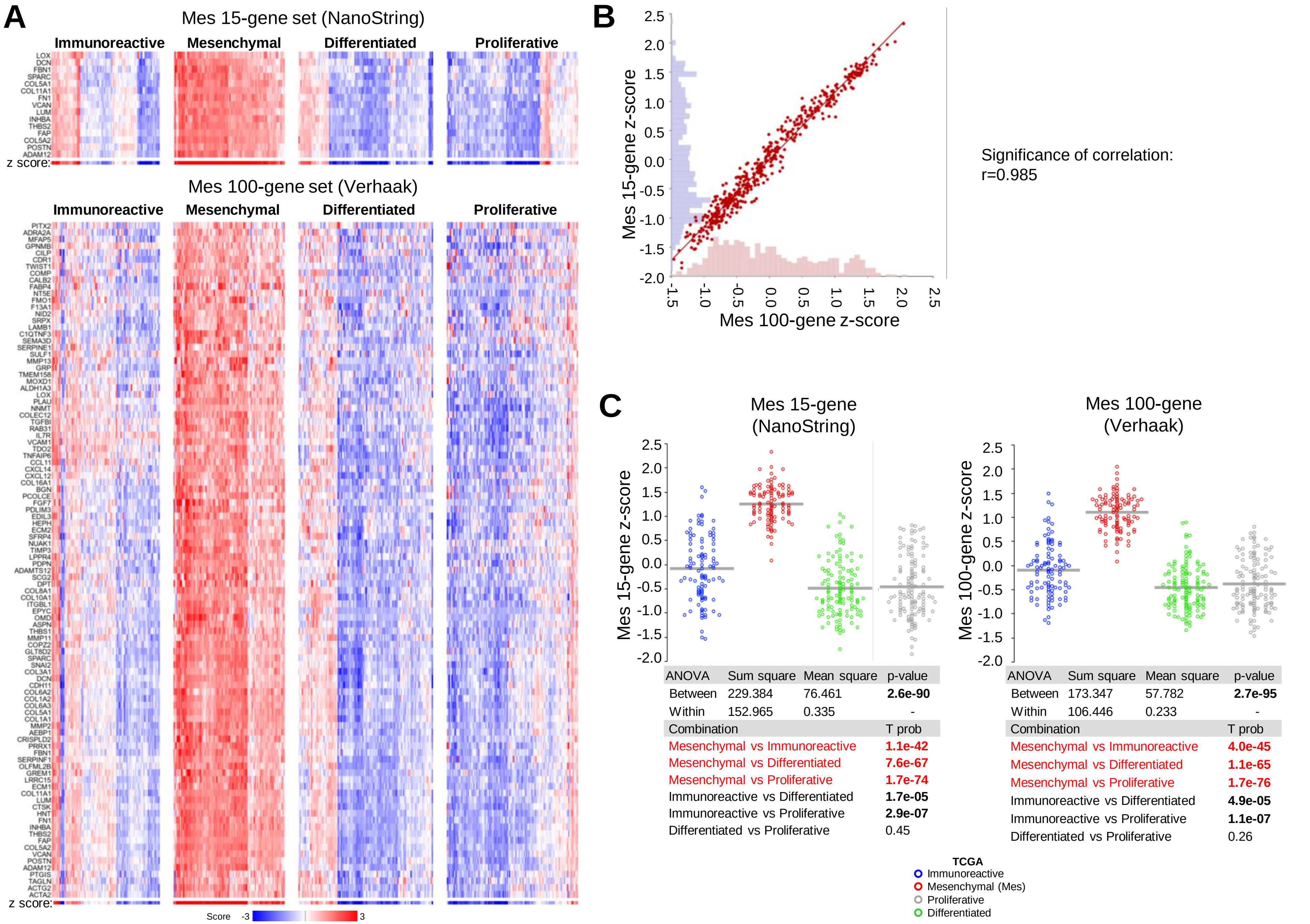

Mes 15-gene (NanoString)

ANOVA	Sum square	Mean square	p-value
Between	229.384	76.461	**2.6e-90**
Within	152.965	0.335	-

Combination	T prob
Mesenchymal vs Immunoreactive	**1.1e-42**
Mesenchymal vs Differentiated	**7.6e-67**
Mesenchymal vs Proliferative	**1.7e-74**
Immunoreactive vs Differentiated	**1.7e-05**
Immunoreactive vs Proliferative	**2.9e-07**
Differentiated vs Proliferative	0.45

Mes 100-gene (Verhaak)

ANOVA	Sum square	Mean square	p-value
Between	173.347	57.782	**2.7e-95**
Within	106.446	0.233	-

Combination	T prob
Mesenchymal vs Immunoreactive	**4.0e-45**
Mesenchymal vs Differentiated	**1.1e-65**
Mesenchymal vs Proliferative	**1.7e-76**
Immunoreactive vs Differentiated	**4.9e-05**
Immunoreactive vs Proliferative	**1.1e-07**
Differentiated vs Proliferative	0.26

Fig. S2

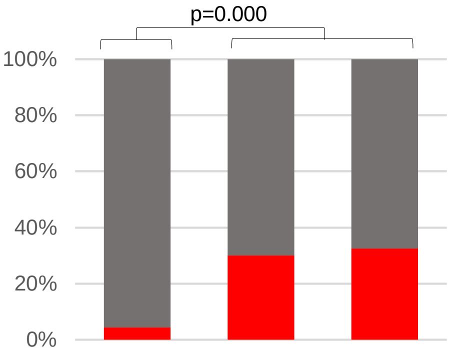

TCGA	ov-ov II	ov-ov III	ov-ov IV
Mes	1	108	24
non-Mes	22	251	50
Total	23	359	74
**% Mes**	**4%**	**30%**	**32%**

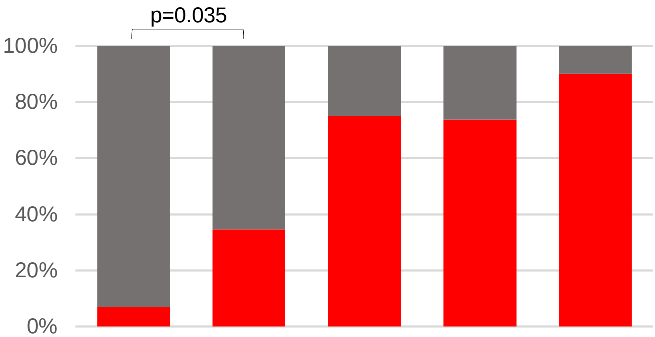

GSE9891	ov-ov I-II	ov-ov III-IV	per-ov II,III,IV	ov-per III-IV	per-per III-IV
Mes	1	36	6	28	18
non-Mes	13	68	2	10	2
Total	14	104	8	38	20
**% Mes**	**7%**	**35%**	**75%**	**74%**	**90%**

Fig. S3

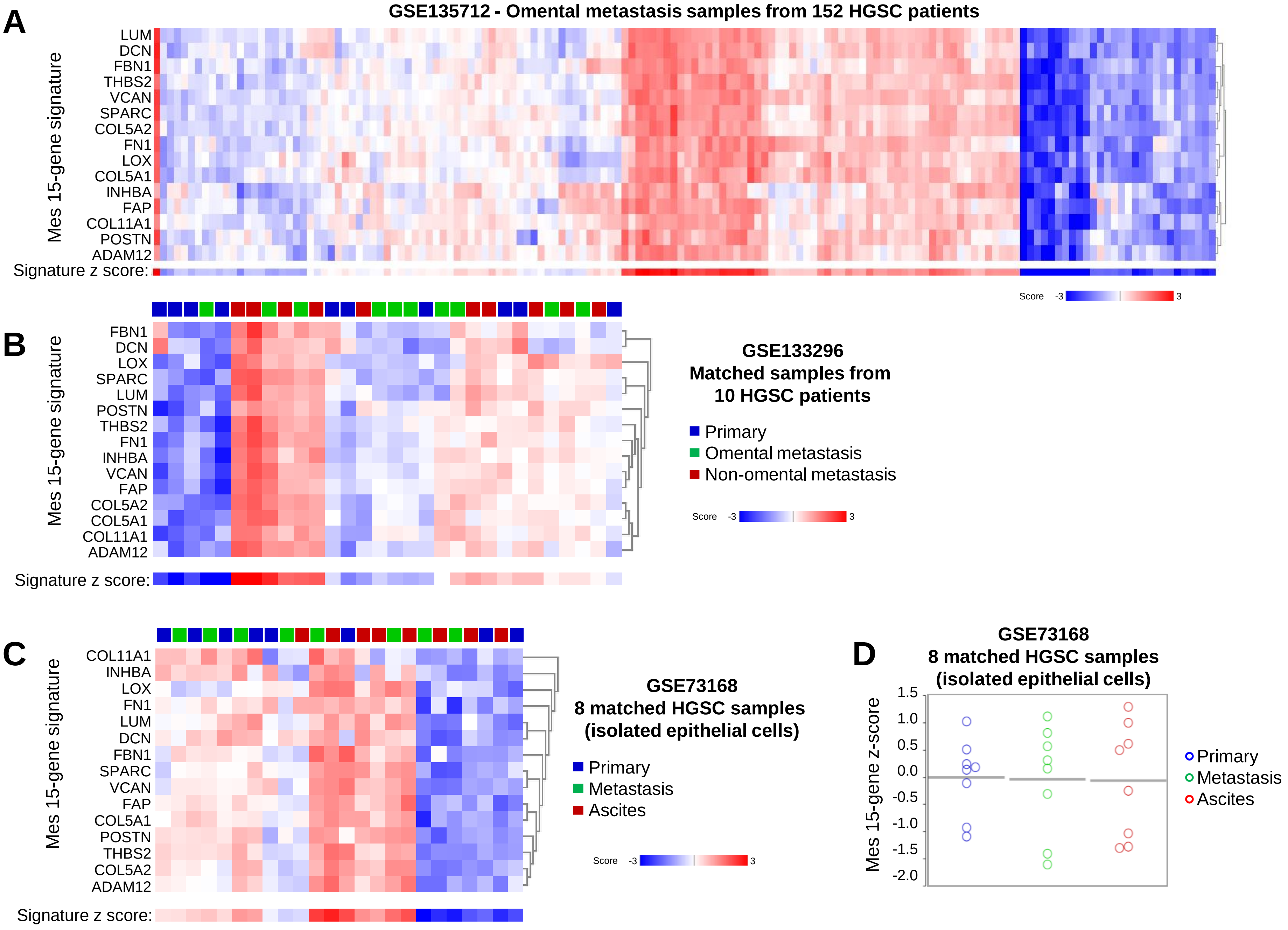
A
GSE135712 - Omental metastasis samples from 152 HGSC patients
Mes 15-gene signature
LUM
DCN
FBN1
THBS2
VCAN
SPARC
COL5A2
FN1
LOX
COL5A1
INHBA
FAP
COL11A1
POSTN
ADAM12
Signature z score:
Score -3 3
B
Mes 15-gene signature
FBN1
DCN
LOX
SPARC
LUM
POSTN
THBS2
FN1
INHBA
VCAN
FAP
COL5A2
COL5A1
COL11A1
ADAM12
Signature z score:
GSE133296
Matched samples from
10 HGSC patients
Primary
Omental metastasis
Non-omental metastasis
Score -3 3
C
Mes 15-gene signature
COL11A1
INHBA
LOX
FN1
LUM
DCN
FBN1
SPARC
VCAN
FAP
COL5A1
POSTN
THBS2
COL5A2
ADAM12
Signature z score:
GSE73168
8 matched HGSC samples
(isolated epithelial cells)
Primary
Metastasis
Ascites
Score -3 3
D
GSE73168
8 matched HGSC samples
(isolated epithelial cells)
Mes 15-gene z-score
1.5
1.0
0.5
0.0
-0.5
-1.0
-1.5
-2.0
Primary
Metastasis
Ascites

Fig. S4
