# Supplementary material for: Are Epithelial Ovarian Cancers of the Mesenchymal Subtype Actually Intraperitoneal Metastases to the Ovary?
Source: Front Cell Dev Biol. 2020 Jul 17;8:647. doi: 10.3389/fcell.2020.00647 (PMC7380132; doi:10.3389/fcell.2020.00647)
Supplement: Supplementary file 2 [file Data_Sheet_2.PDF]

SUPPLEMENTARY FIGURES

Figure S1. 15 Mes genes are expressed in the cancer stroma and associated with poor survival in primary HGSC. **(A)** Expression of the 15 Mes genes in the GSE40595 data set of laser-capture-microdissected stromal and epithelial cancer components from primary HGSC. The number of patients in each group is indicated in parentheses. **(B)** Expression of the 15 Mes genes in the GSE39395 data set of purified cells from fresh colorectal cancer samples using a combination of EPCAM and CD45. The number of patients in each group is indicated in parentheses. **(C)** Kaplan-Meier plot of overall survival and disease-free survival using the 15-gene Mes signature (average expression) in the TCGA data set. Samples include grade 2+3 HGSC. Software used for analysis: Kaplan-Meier Plotter. Gyorffy B, Lanczky A, Szallasi Z. Implementing an online tool for genome-wide validation of survival-associated biomarkers in ovarian-cancer using microarray data of 1287 patients, Endocrine-Related Cancer 2012,19:197-208

Figure S2. Robustness of the Mes 15-gene signature in classifying Mes-HGSC. **(A)** Euclidean clustering heatmaps of expression values of the 15-gene signature defined by NanoString analysis and the 100-gene signature defined by Verhaak et al. in the ovarian TCGA data set in which samples had been previously classified into the Immunoreactive, Mesenchymal, Proliferative and Differentiated molecular subtypes. The signature score was defined as the average z-score of a z-score-transformed TCGA data set. Average gene signature scores are shown at the bottom of each heatmap. **(B)** Spearman correlation of gene signature scores between Mes 15-gene set (NanoString) and Mes 100-gene set (Verhaak). **(C)** Levels of signature scores in the Immunoreactive, Mesenchymal, Proliferative and Differentiated molecular subtypes. The Mes 15-gene signature is equally effective in identifying Mes-HGSC as the standard 100-gene Mes subtype gene set defined by Verhaak et al.

Figure S3. Upper abdominal/omental metastases and PPC exhibit the Mes phenotype while HGSC confined to the pelvis do not. This figure goes with Fig. 2C and D. The Mes 15-gene signature was used for classification of the Mes and non-Mes subtypes instead of the original molecular subtype classification in the TCGA and GSE9891 data sets shown in Fig. 2C and D. The p values indicate the two-tailed Fisher's probability test for the number of Mes and non-Mes samples.

Figure S4. Derivation of the Mes 15-gene z-score in different gene expression data sets. Expression values for the Mes 15-gene signature are shown as heatmaps of Euclidean clustering analysis **(A)** GSE135712, **(B)** GSE133296, and **(C)** GSE73168. Average gene z-scores are shown at the bottom of each heatmap. **(D)** Relative enrichment of the Mes 15-gene z-score in EpCam-positive epithelial cells isolated from primary ovarian HGSC, matched metastases, and ascites samples from 5 patients (3 with duplicate samples). Excluded from the analysis were samples from 3 patients with ovarian tumors of low malignant potential. The bars represent median relative enrichment levels in each group of samples.

SUPPLEMENTARY TABLES

Table S1. Gene expression datasets and associated publications.

Table S2. 15-gene and 100-gene signatures of the Mes molecular subtype. Genes overlapping between the 15-gene and 100-gene signatures are highlighted in yellow.

Table S3. 15-gene signature NanoString mRNA values and algorithm for classifying the Mes subtype in 24 patient-matched primary, metastatic, and recurrent HGSC.

Table S4. Performance of the 15-gene classifier from Table S2 in identifying Mes and non-Mes molecular subtypes in the TCGA data set.

Table S5. Performance of the Mes 15-gene classifier from Table S2 in identifying Mes and non-Mes molecular subtypes in the GSE2109 data set.

Table S6. Metastasis site groups in the GSE2109 data set.
